# Development of early maturing salt-tolerant rice variety KKL(R) 3 using a combination of conventional and molecular breeding approaches

**DOI:** 10.3389/fgene.2023.1332691

**Published:** 2024-02-02

**Authors:** Thirumeni Saminadane, Sathyadevi Geddam, Paramasivam Krishnaswamy, Karthick Jothiganapathy, Anandhan Tamilselvan, Bharathi Raja Ramadoss, Patil Sri Hari Reddy, Uma Shankar Singh, Rakesh Kumar Singh, John Damien Platten, Glenn B. Gregorio, Nagendra Kumar Singh, Deepak Singh Bisht, Suneetha Kota, Senguttuvel Ponnuvel, Padmavathi Guntupalli

**Affiliations:** ^1^ Department of Plant Breeding and Genetics, Pandit Jawaharlal Nehru College of Agriculture and Research Institute, Karaikal, Puducherry, India; ^2^ Centre for Plant Breeding and Genetics, Tamil Nadu Agricultural University, Coimbatore, Tamilnadu, India; ^3^ International Rice Research Institute, Manila, Metro Manila, Philippines; ^4^ College of Agriculture and Food Science, University of the Philippines Los Banos (UPLB), Los Baños, Laguna, Philippines; ^5^ Genomics Laboratory, Indian Council of Agricultural Research (ICAR) - National Institute for Plant Biotechnology, New Delhi, India; ^6^ ICAR - Indian Institute of Rice Research, Hyderabad, Telangana, India

**Keywords:** rice, salinity, seedling-stage salinity tolerance, marker assisted breeding, recombinant inbred line, crop resilience, salt stress adaptation, Saltol QTL

## Abstract

**Introduction:** Soil salinity poses a severe threat to rice production, resulting in stunted growth, leaf damage, and substantial yield losses. This study focuses on developing an early maturing seedling stage salinity tolerant rice variety by integrating conventional breeding methods with marker assisted breeding (MAB) approaches.

**Methods:** Seedling-stage salinity tolerance Quantitative Trait Locus (QTL) “Saltol” from the salt-tolerant parent FL478 was introduced into the high-yielding but salt-sensitive rice variety ADT 45. This was achieved through a combination of conventional breeding and MAB. The breeding process involved rigorous selection, screening, and physiological parameter assessments.

**Results:** KKL(R) 3 (KR 15066) identified as the top performing Recombinant Inbred Line (RIL), consistently demonstrating maximum mean grain yields under both salinity (3435.6 kg/ha) and normal (6421.8 kg/ha) conditions. In comparison to the early maturing, salt-tolerant national check variety CSR 10, KKL(R) 3 exhibited a substantial yield increase over 50%.

**Discussion:** The notable improvement observed in KKL(R) 3 positions it as a promising variety for release, offering a reliable solution to maximize yields, ensure food security, and promote agricultural sustainability in both saline and non-saline environments. The study highlights the effectiveness of MAB in developing salt-tolerant rice varieties and emphasizes the significance of the Saltol QTL in enhancing seedling stage salinity tolerance. The potential release of KKL(R) 3 has the capacity to revolutionize rice production in salt affected regions, providing farmers with a reliable solution to maximize yields and contribute to food security while ensuring agricultural sustainability.

## Introduction

Rice plants are extremely sensitive to salt stress during the seedling and reproductive phases, resulting in a variety with detrimental adverse effects that significantly affect the yield and quality (Changappa et al., 2024). The symptoms of salt damage in rice includes stunted growth, leaf rolling, white ends, drying of older leaves, and pollen sterility (Vinod et al., 2013). The primary causes of these lesions are membrane destabilization, osmotic imbalance, and disruption of the photosynthetic system (Singh et al., 2018). Research findings indicate that for every increase of 1 dS/m beyond the critical salt threshold of 3.0 dS/m, the rice yield decreases by 12%. Overall, soil salinity substantially restricts rice plant growth and development, resulting in significant yield and quality losses, with reductions of over 50% at an EC of 7.2 dS/m being normal (Grieve et al., 2012).

Approximately 5% of India’s cultivated land is affected by saline soil. The majority of these saline soils are found in the Indo-Gangetic Plain, which covers states like Punjab, Haryana, Uttar Pradesh, Bihar, and a portion of Rajasthan. Additionally, arid and semi-arid regions in Gujarat and Rajasthan and dry and semi-arid areas in Gujarat, Madhya Pradesh, Maharashtra, Karnataka, Tamil Nadu, Pondicherry, and Andhra Pradesh also experience the impact of salinity in their soils (Changappa et al., 2024).

Enhancing salt tolerance in rice seedlings through agronomic management strategies can contribute to an increase in crop yield in saline-affected regions. Optimizing irrigation practices, managing soil amendments, and providing a balanced nutrient supply contribute to improving seedling survival and growth in saline conditions. Additionally, the use of growth enhancers, bio-stimulants, and well-planned crop rotation further strengthens seedlings to withstand salt stress. By combining these approaches, farmers can foster healthier rice seedlings, better equipped to cope with salinity, ultimately leading to improved overall crop productivity and food security in regions facing soil salinity challenges. However, there are a number of constraints to consider regarding sustainability, cost, and labor. Therefore, developing salt tolerance in rice seedlings during the early stages of growth is critical for enhancing overall crop production in soil salinity-affected areas sustainably and reducing the cost of cultivation (Shrivastava and Kumar, 2015).

Saltol, a significant quantitative trait locus (QTL) for seedling-stage salinity tolerance in rice, was identified on chromosome 1 in previous investigations (Thomson et al., 2010; Babu et al., 2017a; Singh et al., 2018). This QTL was found to account for 43–70% of the phenotypic variation in the population derived from the IR29 × Pokkali cross for seedling-stage salinity tolerance (Thomson et al., 2010). One specific recombinant inbred line derived from this cross, FL478 (IR66946-3R-178-1-1), which demonstrates salt tolerance during the seedling stage, has been extensively used as a valuable donor for enhancing salt tolerance in rice breeding programs globally. The Saltol area in FL478 has an introgression of a chromosomal fragment of less than 1 megabase (Mb) in size, located within the 10.6–11.5 Mb region, obtained from the salt-tolerant parent Pokkali, according to the haplotype analysis (Kim et al., 2009) Another population resulting from a cross between Nona Bokra and Koshihikari also identified a QTL related to salinity stress in the same genomic region. This QTL was subsequently fine-mapped and cloned as OsHKT1;5, a gene encoding a sodium transporter responsible for maintaining potassium (K^+^) homeostasis (Fan et al., 2021).

In order to mitigate the detrimental impact of salinity on rice cultivation, researchers are currently engaged in the development of enhanced salt-tolerant rice varieties. This is being achieved through the use of conventional breeding techniques (Krishnamurthy et al., 2019a; Krishnamurthy et al., 2019b; Krishnamurthy et al., 2019c), as well as marker assisted breeding (MAB) methods. On the other hand, MAB is a more rapid and precise strategy for transfer of beneficial genes. The use of MAB enables the opportunity to carefully select desired genes during each breeding cycle, ensuring accurate gene transfer. Additionally, MAB offers the advantage of restricting the donor region, thereby mitigating the risk of undesired genetic linkages (Singh et al., 2016).

The application of marker assisted breeding in rice has proven to be effective in the development of a new and improved salt tolerance line in rice, as demonstrated by Linh et al. (2012), Luu et al. (2012), Usatov et al. (2015), Singh et al. (2016), Babu et al. (2017b), Singh et al. (2018), and Bhandari et al. (2019). The use of molecular markers has facilitated the mapping of salt-tolerant genes on rice chromosomes. Several markers, such as RM3412, AP3206, RM8094, RM493, and RM10793, have been identified as being linked to the Saltol genes. These markers have proven useful in marker assisted breeding and screening processes (Ismail et al., 2007; Thomson et al., 2010).

In India, a number of Basmati rice varieties, Pusa Basmati 12; Pusa Basmati 112123; and Pusa Basmati 1509, have been reported to have Saltol QTL introgression, but only limited efforts were made to improve the salinity tolerance of non-Basmati varieties through Saltol introgression. Therefore, there is a need to develop a high-yielding non-Basmati variety for salinity tolerance to stabilize the non-Basmati rice production in salt-affected soils. The rice mega variety ADT 45 is a high-yielding variety under non-saline conditions, but its seedlings are extremely sensitive to salinity stress. Therefore, marker assisted breeding was used to introduce the Saltol QTL into ADT 45 from FL478 in order to develop a new salt-tolerant rice variety with high yields and grain quality traits.

## Materials and methods

### Plant genetic materials and the experimental site

The variety ADT 45, known for its early maturity and high yield but susceptibility to salt, contributes as the female parent, while FL478, possessing salt tolerance, serves as the male parent (Supplementary Table S1). Both parents were first tested for seedling-stage salt tolerance in a hydroponic solution containing an EC of 12 dS/m (105 mM NaCl). The entire greenhouse and field experimentation (GPS coordinates: 10.9504^0^ N and 79.7797^0^ E) was carried out at the Department of Plant Breeding and Genetics, Pandit Jawaharlal Nehru College of Agriculture and Research Institute (PAJANCOA and RI), Karaikal, India.

### Genomic DNA isolation and PCR analysis

The CTAB method was used to extract genomic DNA from the test line leaf tissue (Saghai-Maroof et al. 1984). Following that, PCR amplification was performed using a thermal cycler and a 10 μL reaction mix containing 25–30 ng of genomic DNA and specific primers linked with the Saltol region and foreground selection primers, dNTPs, MgCl_2_, and Taq polymerase. The amplified products of Saltol QTL-linked SSR markers AP3206, RM3412, and RM10793 were then separated on a 3% agarose gel using ethidium bromide and a DNA size standard before being visualized with a UV transilluminator. In the seedling stage of screening and selection, RID 12 (functional marker for the pericarp color) was also used in combination with the Saltol SSR marker to select the white-seeded pericarp. Parental contribution analysis was performed at NIPB, New Delhi-110012, India, on a selected recombinant inbred line (RIL) (F_6_) using a 50 K SNP chip (Singh et al., 2015).

### Crossing and selection of genotypes

ADT 45 was crossed with FL478 to obtain F_1_ seeds, and hybridity was confirmed using Saltol QTL-linked SSR markers AP3206, RM3412, and RM10793. The F_1_ plants were selfed to produce the F_2_ plant. The F_2_ population of 1,212 plants, along with their parents ADT 45, FL478, and IR29 as susceptible checks were evaluated for seedling-stage salinity tolerance using a hydroponic system under greenhouse conditions using the Yoshida nutrient solution (Yoshida, 1976), and 185 plants were scored between 1 and 3. After 1 week, the selected plants were transplanted to a main field under normal conditions to record yield and its component traits. Data on days to flowering (days), plant height (cm), number of tillers per plant, number of grains per panicle, panicle length (cm), 1000 (g), and single plant yield (g) were recorded. Based on the yield performance, 101 plants were selected and screened as F_3_ families and 280 salt-tolerant plants were selected and analyzed for physiological parameters. The number of plants per family selected and screened in each generation is presented in Supplementary Table S2. To identify homozygous plants, the best F_3_ plants were advanced to the F_4_ generation and subjected to foreground selection using all three foreground markers. Additionally, the selected F_4_ plants were progressed to raise F_5_ families, which were tested for their salinity tolerance at the seedling stage. The plants that displayed the highest tolerance to salinity were progressed to the F_6_ generation.

### Screening of RILs against salt stress

The screening process included the use of hydroponics, a method that allowed for precise control of salinity levels at the Department of Plant Breeding and Genetics, Pandit Jawaharlal Nehru Agriculture College and Research Institute, Karaikal, 609603, India. The greenhouse environment is kept at a minimum temperature of 21.2°C–23.8°C, a maximum temperature of 29.2°C–30.4°C, natural sunlight, and a relative humidity of 69%–93%. On the 14th day after sowing, salt stress was administered by adding NaCl to achieve an EC of 12 dS/m in the Yoshida nutritional solution until final scoring. Seedlings were rated after 14 days of salt stress exposure using the rice standard evaluation system (SES) score (IRRI, 2013). The salt injury score of 1 was considered highly tolerant; 3, tolerant; 5, moderately tolerant; 7, susceptible; and 9, highly susceptible.

## Estimation of physiological parameters

### Chlorophyll content

One gram of fresh leaves was pulverized with 20–40 mL of 80% acetone. The pulverized mixture was centrifuged at 5,000–10,000 rpm for 5 min. We collected the supernatant and repeated the extraction until the residue was colorless. The concentrations of chlorophyll a, b, and total were computed using the formula suggested by Arnon (1949) and expressed as mg/g.

### Shoot and root Na^+^, K^+^, and Na^+^/K^+^ ratio

The leaf and root samples were oven-dried (70°C to constant weight) for 72 h, pulverized, and analyzed for the sodium and potassium concentration by flame photometry (ELICO, Hyderabad, India) after 2 hours of extraction with 0.1 N acetic acid, as per the technique described by Thomson et al. (2010). The sample measurements were compared to the standard curve, and the concentration was expressed in millimoles per gram (mmol/g) of dry weight (DW).

### Agronomic performance and grain quality assessment

The agronomic performance of the nine RILs, the parents (ADT 45 and FL478), and CSR 10 (early maturing check variety for salinity) was evaluated with three replications during the main season (Kharif: July–October) of 2020, 2021, and 2022, and the trial was conducted by following the standard package of practices as per the crop production guide 2012, TNAU, Coimbatore (http://agritech.tnau.ac.in/pdf/2013/CPG%202012.pdf), for better crop establishment. Data on days to flowering, plant height, number of tillers per plant, number of grains per panicle, panicle length, 1000 grain weight, and single plant yield were recorded. The plot yield was recorded for each of the replication in kilogram per hectare (kg/ha). The grain and cooking quality traits, such as hulling recovery (HUL), milling recovery (MIL), head rice recovery (HRR), kernel length before cooking (KLBC), kernel breadth before cooking (KBBC), kernel breadth after cooking (KBAC), kernel length after cooking (KLAC), kernel elongation ratio (ER), and alkali spreading value (ASV), were measured according to Jamaloddin et al. (2020).

### Statistical analysis

All of the phenotyping experiments were carried out in three replicates. Data were analyzed using STAR (Statistical Tool for Agricultural Research) version1.10 software developed by the International Rice Research Institute (IRRI)’s Biometrics and Breeding Informatics Group.

## Results

### Response of different filial generation of rice genotypes to Salt Stress

**FIGURE 1:**
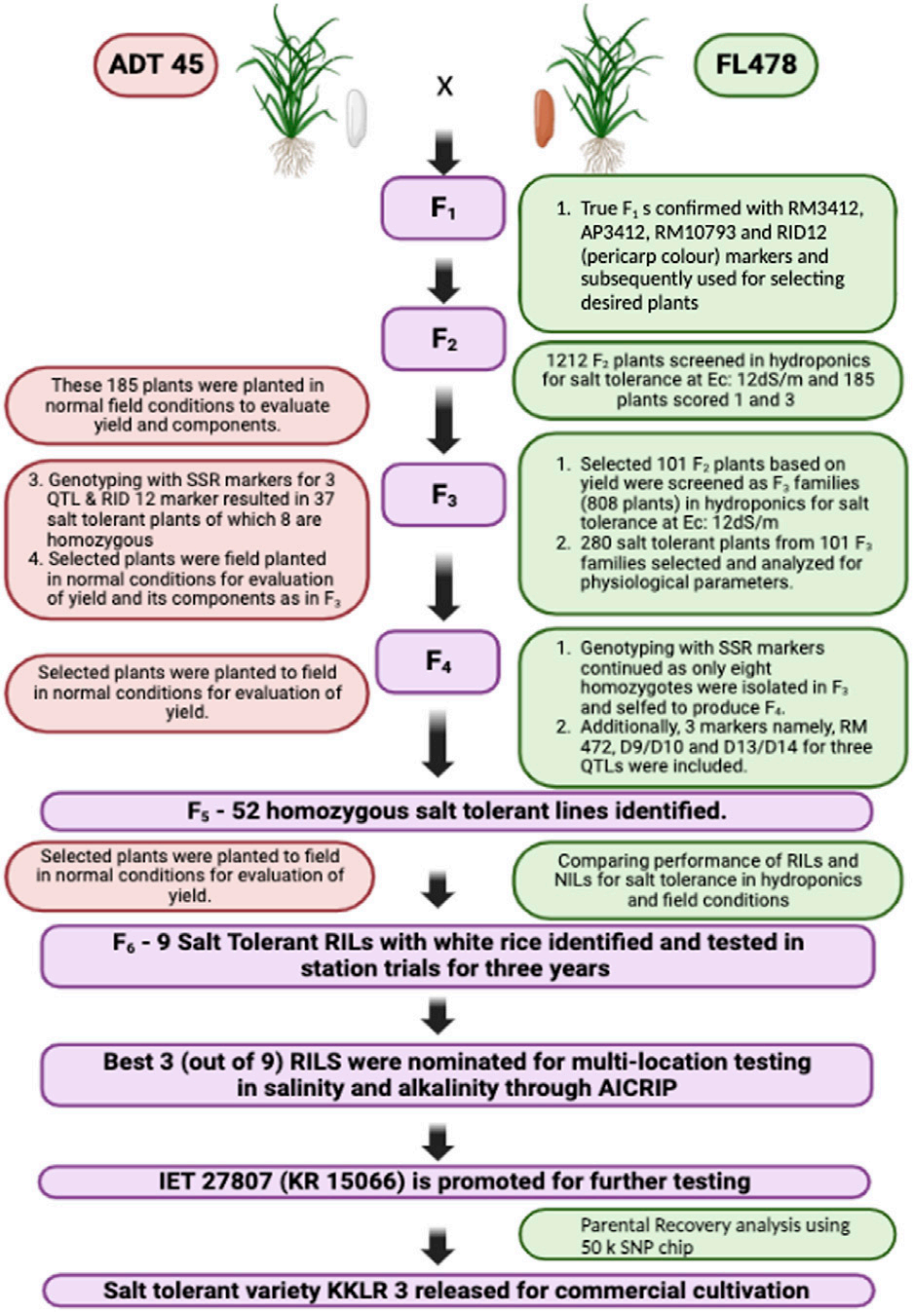
Flowchart showing the combined MAS for development of the early maturing salt tolerant rice variety KKL(R) 3.

**FIGURE 2:**
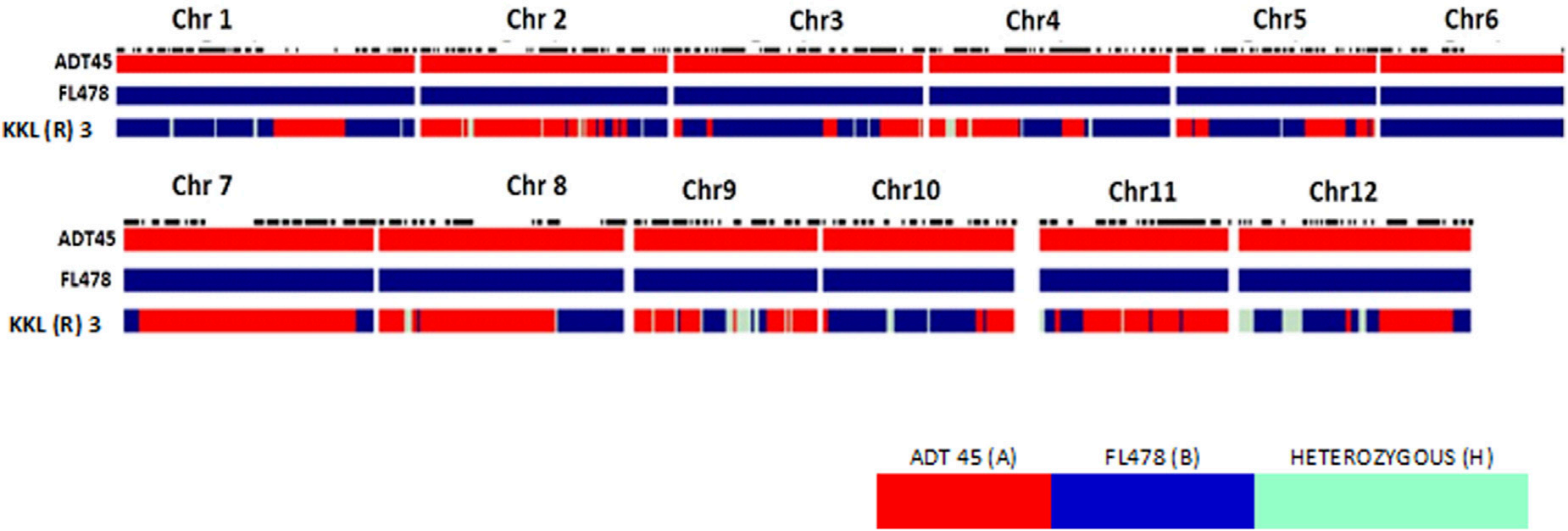
Graphical genotyping of the proposed variety KKL(R) 3 (KR 15066) having desirable characters along with the parents ADT 45 and FL478.

The current study aims to develop the salt tolerance rice variety KKL(R) 3 using the MAB method (Figure 1), under the hydroponic screening condition, with 105 mM NaCl concentration in the hydroponic solution (EC of 12 dS/m). The parents *ADT 45* and *FL478* registered the score of 5 and 1, respectively, on the seventh day of salinization. *ADT 45* registered the score of 9 on the 12th day. To develop a salt-tolerant variety, Saltol was transferred from FL478 to ADT 45 using MAB. A total of 20 true F_1_s were selfed to develop a population of 1,212 F_2_ plants. These F_2_ populations were subsequently subjected to salinity screening in hydroponics to assess their salt tolerance ability. After a thorough screening process, we discovered 185 F_2_ plants that showed an SES score of 1 and 3. The selected F_2_ plants were planted in the normal condition and evaluated for their yield and component traits. A group of 101 F_2_ plants, initially chosen for their yield, underwent subsequent screening to form 808 F_3_ families. Among these, 101 plants were again selected based on their yield performance, while 280 salt-tolerant plants underwent analyses for their physiological parameters. For F_4_ plants, genotyping of the selected tolerant plants was conducted using three Saltol markers and RID 12 markers, yielding 37 salt-tolerant plants, eight of which were identified as homozygous. In F_5_, 52 homozygous salt-tolerant lines were identified; these RILs were screened under the field condition for salt tolerance, and the selected plants were subjected to yield evaluation under the normal condition. Finally, in the F_6_ generation, we identified nine salt-tolerant RILs with white rice and tested them in the station trial for salinity tolerance and yield performance for 3 years. The contribution of the genome from both the parents to the KKL(R) 3 together with the target Saltol QTL is represented graphically in Figure 2.

### Development of breeding lines with Saltol

In each generation, the selected plants were screened for salinity tolerance, and the plants showing an SES score of 1 or 2 were progressed to the next generation. In addition to the SES score, physiological parameters such as chlorophyll content, shoot Na^+^ concentration, shoot K^+^ concentration, shoot Na^+^:K^+^ ratio, root Na^+^ concentration, root K^+^ concentration, and root Na^+^:K^+^ ratio were estimated and taken into account when selecting plants and progressing them to the next generation. Figure 1 illustrates the comprehensive screening and MAS procedure used, as well as the number of plants that were selected and evaluated in every subsequent generation. Table 1 presents an evaluation of the SES scores and other physiological traits of the parents and their populations across several generations. Finally, at the F_6_ generation, nine highly salt tolerant families with exceptional yield and quality attributes were selected and advanced for further testing under the saline and normal field condition.

**TABLE 1:** Mean performance of physiological traits of parents, F_2_, F_3_, F_4_, and F_5_ segregating populations^a^.

Trait	Generation	ADT 45	FL478	ADT 45 × FL478	Selected population
		Mean ± SE	Mean ± SE	Mean ± SE	Mean ± SE
SES score (14th day)	F_2_	8.6 ± 1.2	1.2 ± 0.0	6.9 ± 0.2	1.6 ± 0.9
	F_3_	8.5 ± 1.0	1.3 ± 0.0	5.5 ± 0.1	1.8 ± 0.9
	F_4_	8.2 ± 1.5	1.1 ± 0.0	4.5 ± 0.4	1.5 ± 0.0
	F_5_	8.5 ± 1.2	1.0 ± 0.0	2.8 ± 0.2	1.5 ± 0.0
Chlorophyll content (mg/g)	F_2_	4.1 ± 0.3	6.2 ± 0.2	5.2 ± 0.2	6.6 ± 0.1
	F_3_	4.3 ± 0.2	6.5 ± 0.1	5.4 ± 0.1	6.4 ± 0.1
	F_4_	4.2 ± 0.1	6.4 ± 0.2	5.8 ± 0.2	6.5 + 0.2
	F_5_	4.0 ± 0.1	6.3 ± 0.1	6.0 ± 0.1	6.8 ± 0.1
Shoot Na^+^ concentration (mmol/g dwt)	F_2_	2.0 ± 0.0	0.7 ± 0.0	1.6 ± 0.1	1.5 ± 0.1
	F_3_	2.2 ± 0.1	0.6 ± 0.0	1.8 ± 0.0	1.2 ± 0.0
	F_4_	2.2 ± 0.1	0.6 ± 0.0	1.7 ± 0.1	1.0 ± 0.0
	F_5_	2.1 ± 0.0	0.7 ± 0.0	1.4 ± 0.0	0.8 ± 0.1
Shoot K^+^ concentration (mmol/g dwt)	F_2_	0.4 ± 0.0	0.2 + 0.0	0.3 ± 0.0	0.2 ± 0.0
	F_3_	0.5 ± 0.1	0.2 + 0.0	0.3 ± 0.0	0.2 ± 0.0
	F_4_	0.4 ± 0.0	0.2 + 0.0	0.3 ± 0.0	0.2 ± 0.0
	F_5_	0.4 ± 0.1	0.2 + 0.0	0.3 ± 0.0	0.2 ± 0.0
Shoot Na^+^:K^+^ ratio	F_2_	5.4 ± 0.7	3.5 ± 0.2	4.5 ± 0.3	3.8 ± 0.2
	F_3_	5.1 ± 0.8	3.3 ± 0.2	4.2 ± 0.2	3.6 ± 0.1
	F_4_	5.5 ± 0.4	3.4 ± 0.3	4.0 ± 0.1	3.5 ± 0.2
	F_5_	5.2 ± 0.5	3.4 ± 0.1	4.1 ± 0.1	3.1 ± 0.1
Root Na^+^ concentration (mmol/g dwt)	F_2_	1.4 ± 0.1	0.9 ± 0.1	1.2 ± 0.0	0.8 ± 0.0
	F_3_	1.3 ± 0.1	1.0 ± 0.0	1.1 ± 0.0	0.7 ± 0.0
	F_4_	1.5 ± 0.2	0.8 ± 0.1	1.2 ± 0.0	0.8 ± 0.0
	F_5_	1.3 ± 0.2	0.8 ± 0.1	1.0 ± 0.1	0.7 ± 0.0
Root K^+^ concentration (mmol/g dwt)	F_2_	0.3 ± 0.0	0.1 ± 0.0	0.2 ± 0.0	0.1 ± 0.0
	F_3_	0.2 ± 0.0	0.2 ± 0.0	0.2 ± 0.0	0.1 ± 0.0
	F_4_	0.2 ± 0.0	0.2 ± 0.0	0.2 ± 0.0	0.1 ± 0.0
	F_5_	0.1 ± 0.0	0.1 ± 0.0	0.1 ± 0.0	0.1 ± 0.0
Root Na^+^:K^+^ ratio	F_2_	5.9 ± 0.1	3.9 ± 0.1	5.5 ± 0.1	4.2 ± 0.1
	F_3_	6.0 ± 0.1	3.8 ± 0.0	5.2 ± 0.2	4.0 ± 0.1
	F_4_	5.8 ± 0.1	4.0 ± 0.1	5.3 ± 0.1	3.8 ± 0.1
	F_5_	6.0 ± 0.0	3.9 ± 0.0	5.2 ± 0.4	3.5 ± 0.2

^a^ Characters recorded under saline stress conditions.

### SES and physiological traits

**FIGURE 3:**
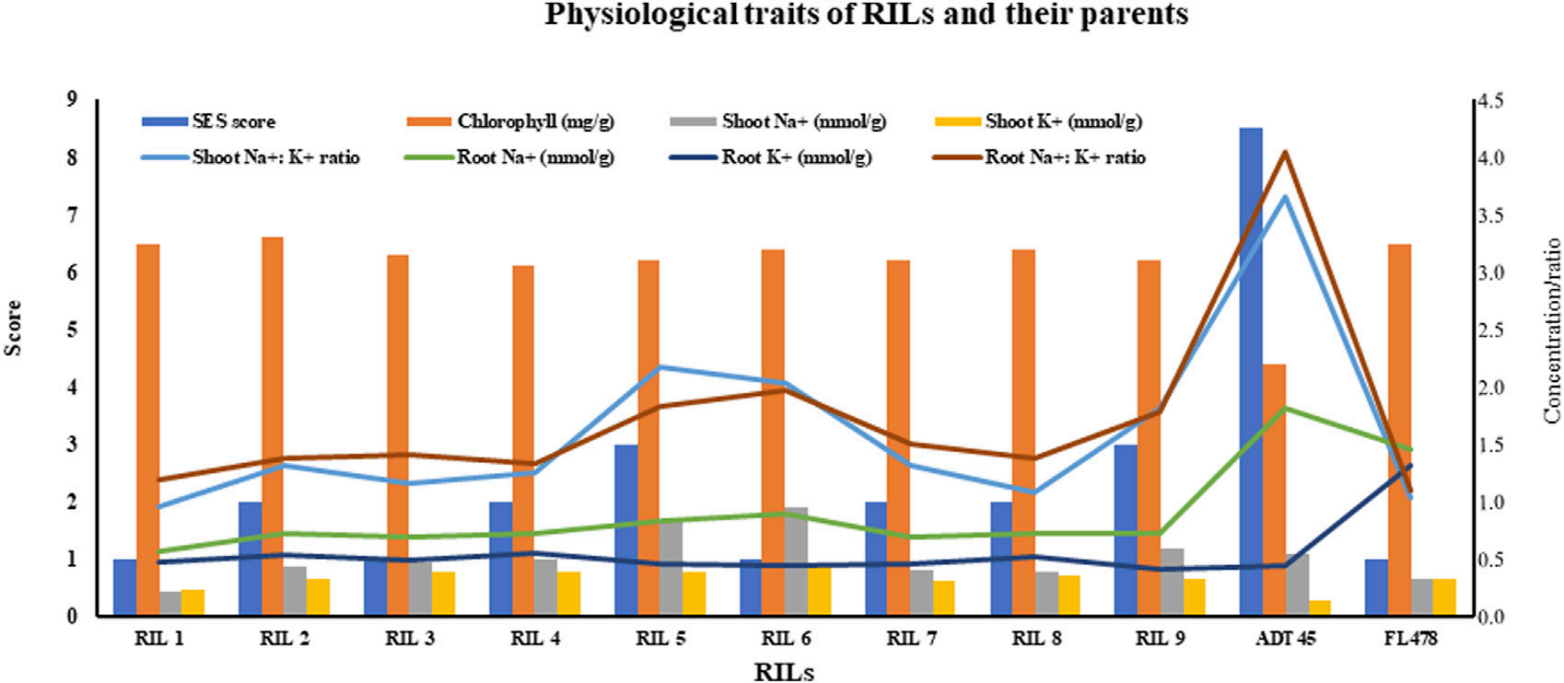
Physiological traits (SES score, chlorophyll, shoot and root Na^+^, shoot and root K^+^, and their ratios) of RILs and parents.

It is important to note that ADT45 and FL478, the parental lines, display significant differences in chlorophyll content, root and shoot sodium and potassium levels, and the ratios compared to the RILs (Figure 3). The SES scores for the RILs range from 1 to 3, while ADT 45 has an SES score of 8.5, and FL478 has an SES score of 1 (Figure 3). RIL 1 stands out as having a low SES score, moderate chlorophyll content, and specific root and shoot sodium and potassium ratios in both the shoots and roots. These characteristics suggest that RIL 1 may have unique adaptations or responses to seedling-stage salinity compared to other RILs.

### Agronomic performance and grain quality of selected lines

The agronomic performance of the selected tolerance lines in each generation was additionally evaluated along with salinity tolerance screening to retain important agronomic traits. The mean performance of RILs for agronomic performance, yield, and its component traits is presented in Table 2. The selected RILs exhibited dissimilarities to both parental lines across the majority of characteristics (Table 3), as selection was carried out towards identifying extreme phenotypes. However, the selection process aimed at selecting white rice as the progeny of FL478, which possesses the property of red rice. Among the RILs analyzed, KR 15066 and KR 15103 were observed to exhibit earlier maturity than the other RILs. Similarly, plant height (PH) exhibited considerable variability, with values ranging from 72.1 to 103.9 cm. Additionally, it was discovered that KR 15066 displayed a significantly higher thousand grain weight (TGW) in comparison to the other RILs. Grain quality attributes, including grain color (GC) and grain type (GT), were also assessed. All the selected RILs exhibited white grains, similar to one of the parent cultivars ADT 45. However, variations in grain type, ranging from long bold to medium slender, were evident, indicating the recombination of different genes.

**TABLE 2:** Agronomic performance, yield, and its component traits of parents, F_2_, F_3_, F_4_, and F_5_ segregating population^a^.

Trait	Generation	ADT 45	FL478	ADT 45 × FL478	Selected population
		Mean ± SE	Mean ± SE	Mean ± SE	Mean ± SE
Days to flowering (days)	F_2_	70.2 ± 0.9	73.3 ± 0.7	73.7 ± 0.2	72.5 ± 1.0
	F_3_	69.3 ± 0.6	74.8 ± 1.0	83.2 ± 0.5	70.0 ± 0.5
	F_4_	72.2 ± 0.8	75.6 ± 1.5	76.2 ± 0.9	68.5 ± 1.0
	F_5_	71.5 ± 0.5	72.4 ± 1.2	80.0 ± 0.8	69.5 ± 0.5
Plant height (cm)	F_2_	92.3 ± 1.4	107.4 ± 1.5	93.2 ± 0.7	92.5 ± 1.2
	F_3_	88.0 ± 2.0	98.1 ± 1.6	92.2 ± 0.6	94.5 ± 2.5
	F_4_	85.3 ± 2.2	100.2 ± 2.4	95.5 ± 1.3	93.5 ± 1.0
	F_5_	90.3 ± 1.6	99.5 ± 1.8	98.0 ± 2.1	90.2 ± 1.5
Number of tillers per plant	F_2_	16.7 ± 1.2	11.4 ± 0.7	15.3 ± 0.3	19.5 ± 1.1
	F_3_	20.2 ± 1.0	12.9 ± 1.0	18.6 ± 0.4	20.2 ± 1.5
	F_4_	18.2 ± 2.4	13.5 ± 1.2	20.1 ± 0.6	22.5 ± 1.0
	F_5_	23.4 ± 1.8	12.4 ± 0.9	22.4 ± 1.4	23.5 ± 1.5
Panicle length (cm)	F_2_	20.6 ± 1.5	24.8 ± 1.6	23.0 ± 3.2	22.5 ± 1.2
	F_3_	22.8 ± 2.4	25.5 ± 2.8	24.7 ± 2.1	23.4 ± 1.0
	F_4_	21.2 ± 1.9	26.2 ± 1.8	25.1 ± 1.4	22.2 ± 0.5
	F_5_	22.2 ± 2.2	25.4 ± 0.4	26.4 ± 1.7	24.5 ± 1.5
Number of grains per panicle	F_2_	142.5 ± 1.9	179.1 ± 2.0	147.5 ± 2.5	160.2 ± 4.5
	F_3_	187.5 ± 9.5	189.8 ± 8.5	191.2 ± 3.0	199.5 ± 6.5
	F_4_	175.0 ± 8.5	185.0 ± 9.5	205.0 ± 9.5	215.5 ± 4.0
	F_5_	182.5 ± 5.5	190.0 ± 7.5	225.0 ± 8.5	235.7 ± 4.5
1,000 grain weight (g)	F_2_	17.1 ± 0.0	28.1 ± 0.1	20.2 ± 0.1	21.2 ± 0.2
	F_3_	17.2 ± 0.0	28.2 ± 0.1	21.3 ± 0.1	22.8 ± 0.5
	F_4_	18.1 ± 0.2	29.5 ± 0.4	22.5 ± 0.4	24.5 ± 1.2
	F_5_	18.5 ± 0.8	28.5 ± 0.5	25.2 ± 0.5	25.6 ± 1.5
Single plant yield (g)	F_2_	29.1 ± 0.5	38.9 ± 0.7	36.9 ± 0.7	38.2 ± 2.4
	F_3_	30.3 ± 1.7	42.3 ± 1.9	42.3 ± 1.2	44.2 ± 1.8
	F_4_	32.4 ± 1.2	40.8 ± 1.7	45.4 ± 1.9	45.6 ± 2.0
	F_5_	30.5 ± 1.5	41.5 ± 1.3	44.7 ± 1.5	45.8 ± 1.2

^a^ Characters recorded under the non-stress condition.

**TABLE 3:** Agronomic performance, yield, maturity duration, salinity score, and grain type of selected RILs in comparison with the parents.

RIL	Culture	DFF	PH	NPT	PL	TGW	SES STS	MD	Y (kg/ha)	GC	GT
RIL 1	KR 15066	90.5 ± 1.0	84.5 ± 2.85	16.5 ± 0.75	23.4 ± 1.5	25.95 ± 0.40	1	107	3530.75 ± 101.84	White	Long bold
RIL 2	KR 15075	108 ± 2.0	87.5 ± 1.57	20.2 ± 1.25	20.4 ± 2.1	21.65 ± 0.72	2	126	4170.37 ± 284.38	White	Medium slender
RIL 3	KR 15076	93 ± 1.0	99.3 ± 2.59	18.4 ± 0.25	22.5 ± 0.9	24.90 ± 0.52	1	112	4130.69 ± 167.25	White	Long slender
RIL 4	KR 15077	102 ± 1.0	72.1 ± 4.18	19.5 ± 0.95	21.8 ± 1.8	18.80 ± 1.2	2	121	3720.50 ± 124.17	White	Medium slender
RIL 5	KR 15083	105 ± 2.0	90.4 ± 3.65	16.5 ± 1.25	20.9 ± 3.1	17.80 ± 1.5	3	121	3440.25 ± 114.10	White	Medium slender
RIL 6	KR 15092	103 ± 1.0	98.5 ± 1.18	17.2 ± 2.50	19.5 ± 2.2	20.50 ± 0.8	1	124	3690.18 ± 151.37	White	Medium slender
RIL 7	KR 15100	108 ± 1.0	103.9 ± 4.36	19.8 ± 0.50	18.9 ± 1.4	18.43 ± 1.1	2	123	4000.85 ± 204.91	White	Medium slender
RIL 8	KR 15101	98 ± 1.0	93.7 ± 3.05	17.5 ± 0.75	25.1 ± 1.2	18.25 ± 0.90	2	120	4840.31 ± 299.24	White	Medium slender
RIL 9	KR 15103	85 ± 2.0	88.9 ± 1.25	16.5 ± 1.50	23.4 ± 2.8	22.20 ± .56	3	103	3660.72 ± 205.87	White	Long slender
ADT 45	-	81 ± 2.0	90.4 ± 1.62	20.4 ± 0.75	21.5 ± 2.1	17.50 ± 0.84	8.5	118	3430.34 ± 145.71	White	Medium slender
FL478	-	94 ± 1.0	102.5 ± 1.55	13.2 ± 0.50	25.2 ± 1.9	25.90 ± 0.73	1	125	4260.67 ± 220.63	Red	Long bold

DFF, days to 50% flowering; PH, plant height in cm; NT, number of productive tillers; PL, panicle length in cm; TGW, thousand grain weight in g; SES STS, standard evaluation salt tolerance score under field condition (micro-plot); MD, maturity duration; Y, yield kg/ha; GC, grain color; GT, grain type.

**FIGURE 4:**
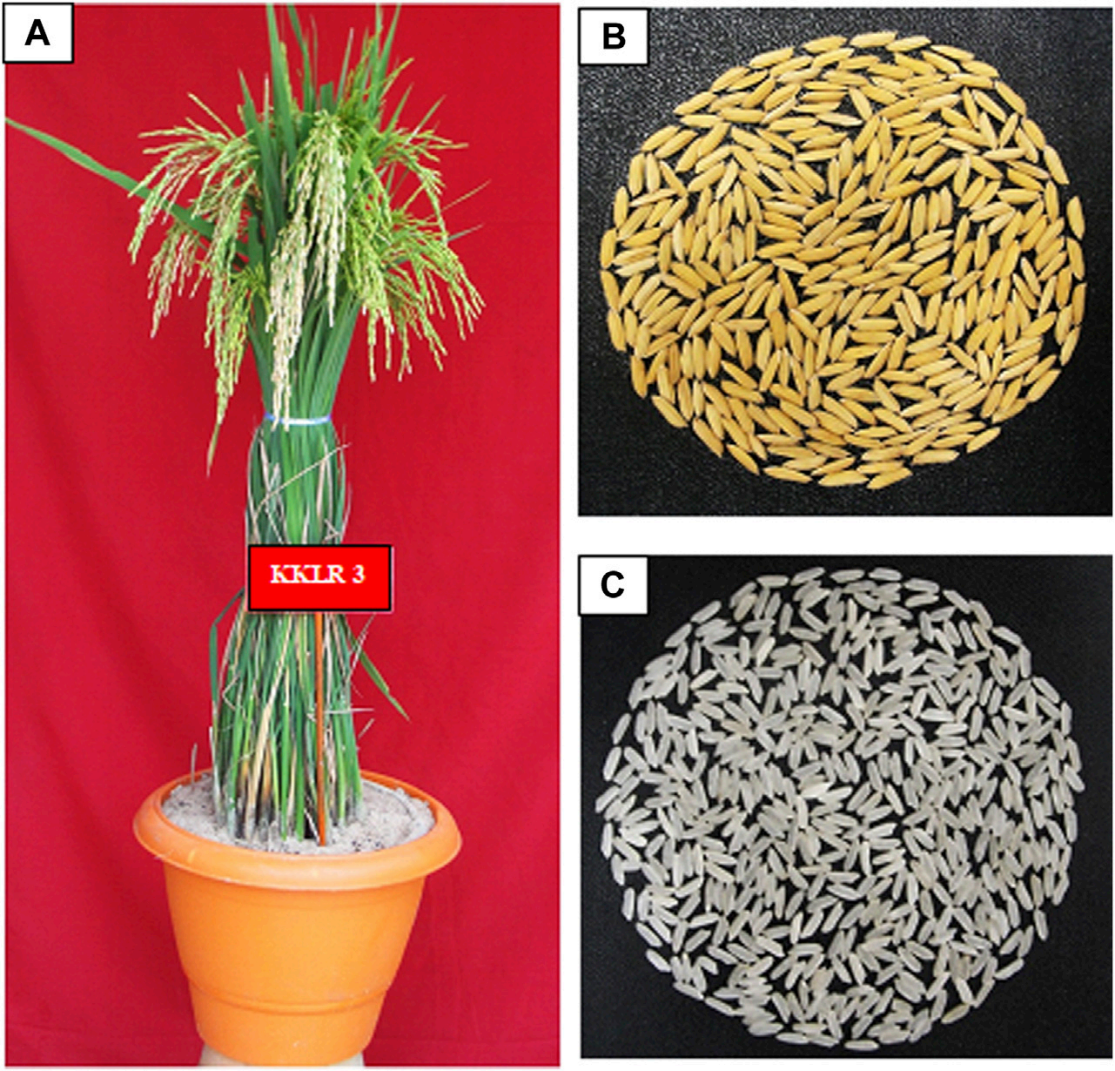
Morphology of the salt-tolerant rice variety KKL(R) 3. **(A)** Single plant, **(B)** paddy, and **(C)** rice.

The mean grain and cooking quality characteristics of RILs along with their parents are presented in Table 4. The hulling percentage and milling percentage are key indicators of grain processing efficiency. The RILs displayed varying HUL and MIL values, with the HUL value ranging from 75.8% to 80.4% and the MIL value ranging from 64.5% to 70.6%. RIL 2 and 5 recorded the lowest and highest HRR of 54.6% and 60.2%, respectively. RIL 1 (Figure 4), RIL 2, RIL 3, and RIL 8 showed more than 27% of amylose content. However, amylose content is highly similar to one of the parents, ADT 45. Except for RIL 4, RIL 5, and RIL 8, all other RILs possessed the alkali spreading value of 5, similar to that of ADT 45.

**TABLE 4:** Grain quality traits of RILs in comparison with ADT 45 and FL478.

RIL	Culture	HUL	MIL	HRR	KLBC	KBBC	ASV	Amylose (%)	GC
RIL 1	KR 15066	76.5 ± 1.8	66.4 ± 2.1	58.2 ± 2.3	6.25 ± 0.24	2.26 ± 0.07	5	27.54 ± 1.5	40.5 ± 2.5
RIL 2	KR 15075	80.4 ± 3.4	70.6 ± 1.7	54.6 ± 1.3	6.01 ± 0.18	2.18 ± 0.10	5	27.07 ± 2.1	41.5 ± 2.1
RIL 3	KR 15076	78.6 ± 2.7	64.5 ± 1.1	59.4 ± 1.6	6.58 ± 0.31	2.29 ± 0.08	5	27.28 ± 1.1	43.2 ± 1.9
RIL 4	KR 15077	75.8 ± 2.4	68.3 ± 2.2	55.6 ± 3.1	6.46 ± 0.28	2.08 ± 0.09	4	26.69 ± 1.8	41.5 ± 1.5
RIL 5	KR 15083	79.3 ± 1.0	66.2 ± 3.1	60.2 ± 2.7	6.74 ± 0.19	2.19 ± 0.14	4	25.68 ± 1.4	40.4 ± 1.8
RIL 6	KR 15092	77.5 ± 1.5	69.4 ± 2.8	59.1 ± 0.9	6.55 ± 0.25	2.05 ± 0.11	5	25.73 ± 1.2	41.5 ± 2.0
RIL 7	KR 15100	78.9 ± 2.8	69.1 ± 1.6	57.4 ± 1.8	6.13 ± 0.31	2.16 ± 0.09	5	25.16 ± 0.9	40.5 ± 1.8
RIL 8	KR 15101	80.2 ± 0.9	68.9 ± 2.5	56.9 ± 1.6	6.64 ± 0.27	2.24 ± 0.13	4	27.49 ± 1.0	42.5 ± 2.2
RIL 9	KR 15103	79.5 ± 2.1	65.7 ± 2.9	55.4 ± 2.2	6.87 ± 0.16	2.14 ± 0.14	5	26.47 ± 1.5	43.5 ± 1.9
ADT 45	-	78.5 ± 1.6	65.2 ± 1.9	59.2 ± 1.4	5.64 ± 0.21	1.92 ± 0.15	5	26.44 ± 1.3	41.3 ± 2.2
FL478	-	72.9 ± 1.3	63.5 ± 1.7	52.4 ± 1.2	7.61 ± 0.25	2.30 ± 0.03	5	23.65 ± 1.4	42.5 ± 1.9

HUL, hulling percentage; MIL, milling percentage; HRR, head rice recovery in percentage; KLBC, kernel length before cooking in mm; KBBC, kernel breadth before cooking in mm; ASV, alkali spreading value; GC, gel consistency in mm.

### Evaluation of the selected lines under multi-location trials

The yield data for the parents and all the recombinant inbred lines were assessed for three successive Kharif seasons from 2020 to 2022 under saline and normal conditions (Table 5). In general, all RILs experienced a reduction in grain yield under saline conditions, reflecting the adverse effects of salinity on rice. Under saline conditions, the grain yield of the RILs exhibited a noticeable variability across the three Kharif seasons. RIL 1 (KR 15066) displayed the highest mean grain yield of 3435.6 ± 286.3 kg/ha, followed closely by RIL 2 (KR 15075) and RIL 3 (KR 15076) with mean grain yields of 3133.6 ± 182.3 kg/ha and 2998.6 ± 191.6 kg/ha, respectively. In contrast, RIL 4 (KR 15077) exhibited a lower mean grain yield of 2749.3 ± 243.8 kg/ha, indicating lower salt tolerance. Under normal conditions, RIL 1 (KR 15066) maintained its superior performance, with a mean grain yield of 6421.8 ± 174.2 kg/ha, which was significantly higher than that of the other RILs. RIL 7 (KR 15100) and RIL 2 (KR 15075) also exhibited higher mean grain yields under normal conditions, further highlighting their adaptability to varying environmental conditions. Considering KR 15066 (KKL(R) 3’s consistent and superior performance in salinity conditions during the Kharif season in 2020–2022, KKL(R) 3 demonstrated a yield increase of approximately 16% to 50% (Table 5) over the early maturing national check variety CSR 10.

**TABLE 5:** Yield performance of RILs in comparison with the parents across three cropping seasons under the saline and normal condition.

RIL	Culture	Grain yield (kg/ha) under saline condition				Grain yield (kg/ha) under normal condition			
		Kharif 2020	Kharif 2021	Kharif 2022	Mean	Kharif 2020	Kharif 2021	Kharif 2022	Mean
RIL 1	KR 15066	3204.5 ± 354.9	3471.8 ± 203.8	3630.4 ± 300.2	3435.6 ± 286.3	7145.5 ± 194.5	6058.3 ± 197.7	6061.8 ± 130.4	6421.8 ± 174.2
RIL 2	KR 15075	3032.1 ± 158.4	3126.7 ± 237.8	3241.9 ± 150.6	3133.6 ± 182.3	5824.2 ± 246.0	5958.5 ± 123.4	6001.1 ± 249.5	5927.9 ± 206.3
RIL 3	KR 15076	2964.7 ± 136.8	3031.4 ± 242.6	2999.7 ± 195.5	2998.6 ± 191.6	5791.7 ± 156.4	5542.3 ± 186.3	5634.1 ± 185.4	5656.0 ± 176.0
RIL 4	KR 15077	2845.6 ± 292.7	2657.4 ± 164.2	2744.8 ± 274.5	2749.3 ± 243.8	5312.4 ± 101.5	5225.3 ± 374.5	5542.6 ± 228.9	5360.1 ± 234.9
RIL 5	KR 15083	2974.2 ± 199.5	2856.7 ± 225.4	2798.4 ± 173.2	2938.9 ± 199.4	6001.5 ± 253.6	5895.7 ± 213.2	5674.2 ± 295.7	5857.1 ± 254.2
RIL 6	KR 15092	3001.5 ± 154.6	2874.6 ± 203.1	2945.7 ± 149.4	2940.6 ± 169.0	5512.6 ± 345.2	5824.9 ± 295.8	5726.1 ± 167.3	5687.9 ± 269.4
RIL 7	KR 15100	2836.7 ± 395.4	2648.4 ± 188.4	2967.9 ± 169.4	2817.6 ± 251.0	6058.4 ± 154.7	5974.8 ± 107.5	5884.9 ± 165.8	5972.7 ± 142.6
RIL 8	KR 15101	2749.3 ± 265.4	2895.1 ± 216.4	3007.2 ± 173.2	2883.8 ± 218.3	5764.5 ± 249.7	5943.2 ± 264.8	5841.3 ± 194.5	5849.7 ± 236.3
RIL 9	KR 15103	2845.6 ± 161.4	2791.5 ± 149.5	3154.2 ± 214.7	2930.4 ± 175.2	5561.2 ± 158.6	5867.8 ± 268.2	5642.6 ± 116.4	5690.5 ± 181.0
ADT 45	-	1767.4 ± 235.7	1829.2 ± 243.6	1789.3 ± 187.6	1795.3 ± 222.3	5262.7 ± 265.4	5337.3 ± 239.5	5245.1 ± 147.2	5281.7 ± 217.3
FL478	-	2694.3 ± 239.6	2769.3 ± 196.2	2994.2 ± 203.5	2819.2 ± 213.1	3542.5 ± 169.2	3778.6 ± 213.9	3998.7 ± 195.5	3773.3 ± 192.9
CSR 10 (check)		2742.5 ± 114.2	2840.3 ± 152.4	2367.8 ± 203.6	2650.3 ± 156.7	-	-	-	-

CSR 10 (check)—check variety to compare the yield under the salinity condition.

## Discussion

### Development of salt tolerant RILs

The development of salt-tolerant rice varieties is critical for mitigating the adverse effects of soil salinity on crop production. This study demonstrates the potential of MAB as an effective strategy for introducing salt tolerance traits into high-yielding rice varieties. Salinity stress can significantly impact rice grain yield and quality, with reductions ranging from 20% to 100%, depending on the severity and duration of exposure to saline conditions (Krishnamurthy et al., 2016a). The extensive genetic diversity among rice varieties in response to soil salinity affords the opportunity to develop salt-tolerant rice cultivars (Akbar et al., 1972; Krishnamurthy et al., 2016b; Krishnamurthy et al., 2019a; Krishnamurthy et al., 2019b; Krishnamurthy et al., 2019c). The susceptible parent ADT 45 is a widely cultivated high-yielding rice variety in India, known for its excellent performance under normal growing conditions. It has gained popularity among farmers due to its high grain yield and grain quality attributes. However, ADT 45 is highly susceptible to salinity stress during the seedling stage. When exposed to elevated levels of soil salinity, ADT 45 experiences adverse effects, including stunted growth, reduced tillering, leaf rolling, and a decline in overall vigor. Its seedlings are extremely sensitive to salt stress, making it challenging to grow in regions with salinity-affected soils. The tolerant cultivar FL478 exhibits remarkable tolerance to salinity stress, particularly during the seedling stage. The salt tolerance of FL478 is attributed to the presence of the Saltol QTL on chromosome 1. This QTL, which contains key salt tolerance genes like OsHKT1;5, SOS1, SOS2, SOS3, NHX (Na^+^/H^+^ exchanger) family genes, and HAK/KUP/KT family potassium transporters, plays a crucial role in regulating ion transport, osmotic balance, and stress responses, enabling FL478 to adapt and perform well under saline conditions. The area of ADT 45, the chosen mega variety under cultivation, has been reduced by farmers due to increasing soil salinity. The efforts are ongoing to generate salt-tolerant cultivars using the conventional breeding methods. However, MAB allows for the selection of specific genes or traits of interest with high precision, enabling breeders to speed up the breeding process and reduce the need for extensive field trials. This targeted approach minimizes the acquisition of unwanted traits, reducing the risk of linkage drag and maintaining the desired genetic background of the parent variety. Several researchers have successfully incorporated the Saltol QTL into various rice varieties, including Luu et al. (2012) in AS996, Babu et al. (2017b) in PB1121, Geetha et al. (2017) in ADT 43, Krishnamurthy et al. (2020) in Pusa 44 and Sarjoo 52, and Yadav et al. (2020) in PB 1509. By targeting the Saltol QTL, which plays a significant role in seedling-stage salinity tolerance, this research leverages the power of molecular markers in accelerating the breeding process. Therefore, in this study, we developed a seedling-stage salinity-tolerant rice variety KKL(R) 3 through the transfer of the Saltol QTL from FL478 to ADT 45 using MAB.

### SES and physiological traits

The SES scores recorded on the 14th day offer valuable insights into the general salt tolerance of rice plants. Throughout generations (from F_2_ to F_5_), we notice a consistent decrease in SES scores with higher salt concentrations (days under salt stress), indicating a progressive decline in plant tolerance. Chlorophyll content is another crucial parameter for assessing salt tolerance, as it reflects a plant’s ability to maintain photosynthetic activity under salt stress. During each generation, in addition to the SES score, the segregated plants were selected based on chlorophyll content, shoot Na^+^, shoot K^+^, root Na^+^, and root K^+^ along with their ratios. Similarly, the Basmati rice variety PB 1509 was improved by transferring Saltol QTL from FL478, which led to the development of improved PB 1509 with tolerance to salinity stress through marker-assisted backcross breeding (MABB). Similarly, seedling-stage salinity tolerance was improved in Pusa Basmati 1121 through MAS (Babu et al., 2017a). Researchers used similar screening and physiological factors in all of these studies to develop a rice line resistant/tolerant to seedling-stage tolerance (Luu et al., 2012; Babu et al., 2017b; Krishnamurthy et al., 2020).

### Saltol QTL genes in enhancing salinity tolerance in RILs

The MAS was critical in this study since it accelerated the breeding process. MAS enabled for the identification of a particular genomic region associated with the Saltol QTL by using three markers that are closely linked to the Saltol QTL; subsequent MAS in each generation assisted in identifying the plants harboring the Saltol QTL. The Saltol QTL, located on rice chromosome 1, is pivotal for seedling-stage salinity tolerance due to its multiple contributing genes. OsHKT1;5 within this QTL selectively absorbs essential potassium ions while excluding excess sodium ions, preventing their toxic accumulation in plant tissues. SOS1, another crucial gene in the Saltol QTL, pumps out excess sodium ions from plant cells, maintaining cellular ion balance. Recent research supports OsHKT1;5 overexpression in FL478 and related lines under salinity stress, suggesting its role in seedling-stage tolerance. Additionally, SOS1 prevents sodium buildup, ensuring cellular homeostasis. The SOS2–SOS3 complex responds to high salt levels by activating ion transporters and stress-responsive genes. NHX genes sequester excess sodium ions in vacuoles, reducing their impact on cellular health. Meanwhile, HAK/KUP/KT transporters regulate potassium uptake, aiding in ion balance against sodium. Together, these Saltol QTL genes regulate ion transport, osmotic balance, and stress responses. This study successfully transferred Saltol from FL478 to ADT 45, generating nine salt-tolerant RILs with balanced ion levels under stress, crucial during the sensitive seedling stage.

### Selection of the KKL(R) 3 rice variety through multi-season evaluation

The rice RILs developed in this study hold immense promise for farmers in salt-affected areas, offering them the opportunity to harness the full yield potential even in salt-affected soils. Among the RILs, KR 15066 (RIL 1) emerged as a standout performer in terms of salt tolerance. It consistently demonstrated the highest mean grain yield of 3435.6 ± 286.3 kg/ha under saline conditions, demonstrating its remarkable ability to maintain productivity even in the face of salinity stress. This consistent performance across multiple seasons underscores the robustness of KR 15066 as a potential salt-tolerant variety. Furthermore, under normal conditions, KR 15066 (RIL 1) continued to stand out, with a mean grain yield of 6421.8 ± 174.2 kg/ha, significantly surpassing the other RILs. This dual adaptability of KR 15066 (KKL(R) 3) to both saline and normal conditions positions it as a promising candidate for release as a salt-tolerant rice variety.

## Conclusion

In summary, the consistent and remarkable performance of KKL(R) 3 (KR 15066) under both saline and normal conditions makes it a strong candidate for release as a salt-tolerant rice variety. Its potential to thrive in salt-affected soils offers a promising solution for farmers in regions facing salinity challenges, contributing to increased rice yields and food security in the south eastern part of the Indian sub-continent. Further research and field trials will be essential in validating and harnessing the full potential of KKL(R) 3 (KR 15066) for widespread adoption in agricultural practices.

## Data Availability

The original contributions presented in the study are included in the article/Supplementary Material; further inquiries can be directed to the corresponding authors.
