# Supplementary material for: Development of early maturing salt-tolerant rice variety KKL(R) 3 using a combination of conventional and molecular breeding approaches
Source: Front Genet. 2024 Feb 2;14:1332691. doi: 10.3389/fgene.2023.1332691 (PMC10869446; doi:10.3389/fgene.2023.1332691)
Supplement: Supplementary file 1 [file Table1.DOCX]

**Supplementary Table 1. Details of KKL (R) 3 and its parent**

| **Sl.no** | **Characters/ parents** | ***ADT45*** | ***FL478*** | ***KKL (R) 3*** |
| --- | --- | --- | --- | --- |
| 1 | **Parentage** | *ADT37/IR50* | *IR29/Pokkali* | *ADT 45/FL478* |
| 2 | **Type of germplasm** | Cultivar | Breeding line | Recombinant Inbred Line |
| 3 | **Station** | TRRI, Aduthurai | IRRI, Philippines | PAJANCOA & RI, Karaikal. |
| 4 | **Season** | Kuruvai (June- October) | **-** | Kuruvai ((June- October) |
| 5 | **Duration(days)** | 105 - 110 | 110 - 115 | 110 - 115 |
| 6 | **Plant height (cm)** | 95 | 108 | 90 |
| 7 | **1000 grain weight(gm)** | 17.5 | 28.2 | 27.06 |
| 8 | **Kernel length (mm)** | 5.0 | 8.0 | 9.6 |
| 9 | **Kernel Breadth (mm)** | 2.0 | 2.5 | 2.6 |
| 10 | **Kernel LB ratio** | 2.50 | 3.20 | 3.13 |
| 11 | **Rice grade** | Medium slender | Long slender | Long Bold |
| 12 | **Rice colour** | White | Red | White |
| 13 | **Tolerance to salt stress** | Susceptible | Highly tolerant | Highly tolerant |
| 14 | **Ancestors** | Pachiam, Marong Paroc  Konamani, Sinapwagh  Benong, Cina, Latisail  Vellaikar, Dee-gee-woo-gen  Tsi-yuan chung, Tadukan  Gampai, *O.nivara*, W1543  Unknown *indica,* Arikkiri  Unknown*Japanese*,Thevvan  Vellaiillankalyan Pachoperumal  Eravapandi | Pachiam, Marong Paroc  Konamani, Sinapwagh  Benong, Cina, Latisail  Vellaikar, Dee-gee-woo-gen  Tsi-yuan chung, Tadukan  Gampai, *O.nivara*  Unknown *indica*  Unknown *Japanese, Pokkali* | Pachiam, Marong Paroc  Konamani, Sinapwagh  Benong, Cina, Latisail  Vellaikar, Dee-gee-woo-gen  Tsi-yuan chung, Tadukan  Gampai, *O.nivara,* Arikkiri  Unknown *indica,* W1543  Unknown *Japanese, Pokkali,* Thevvan Vellaiillankalyan, Pachoperumal Eravapandi |

**TRRI** - Tamilnadu Rice Research Institute

**IRRI** - International Rice Research Institute

**PAJANCOA & RI** – Pandit Jawaharlal Nehru College of Agriculture and Research Institute,
