# Supplementary material for: Development of early maturing salt-tolerant rice variety KKL(R) 3 using a combination of conventional and molecular breeding approaches
Source: Front Genet. 2024 Feb 2;14:1332691. doi: 10.3389/fgene.2023.1332691 (PMC10869446; doi:10.3389/fgene.2023.1332691)
Supplement: Supplementary file 2 [file Table2.DOCX]

**Supplementary Table 2.** Number of selected individual/families in each generation.

| **Filial Generation** | **Total Population** | **Selection** |
| --- | --- | --- |
| F_2_ | 1212 | 185 |
| In F_2_, the selection process relies on individual plants. However, starting from the F_3_ generation and onwards, the selection is family-oriented and involves the use of molecular markers. | | |
| F_3_ | 101 | 280 |
| F_4_ | 8 | 52 |
| F_5_ | 52 | 9 |
| F_6_ | 9 | Nine RILS were chosen, evaluated further in a multi-location study, and then one of the RIL (KR 15066) was released as KKL (R) 3 |
